# Pedigree-based evaluation of classical, ancestral, and new inbreeding coefficients in Carora and Criollo Limonero dairy cattle populations

**DOI:** 10.14202/vetworld.2026.3385-3400

**Published:** 2026-08-08

**Authors:** Luis F. Cartuche-Macas, José R. Perez-Gonzalez, Ernesto J. Jimenez Quintero, Anixon G. Leal-González, Joar Garcia-Flores, Manuel Garcia-Herreros

**Affiliations:** 1 Escuela Superior Politécnica Agropecuaria de Manabí Manuel Félix López (ESPAM), Carrera de Medicina Veterinaria, Calceta 130602, Ecuador.; 2 Universidad Politécnica Territorial de Maracaibo (UPTM), Maracaibo 4001, Venezuela.; 3 Asociación de Criadores de Ganado Carora (ASOCRICA), Barinas 5501, Venezuela.; 4 Estación Local Carrasquero, Hacienda El Laral, Instituto Nacional de Investigaciones Agrícolas (INIA), Carrasquero 4044, Venezuela.; 5 Facultad de Ciencias Veterinarias y Agronomía, Universidad UTE, Quito 170147, Ecuador.; 6 National Institute for Agricultural and Veterinary Research (INIAV), Santarém 2005-424, Portugal.; 7 CIISA-AL4AnimalS, Faculty of Veterinary Medicine, University of Lisbon, Lisbon 1300-477, Portugal.

**Keywords:** ancestral inbreeding, Carora cattle, conservation genetics, Criollo Limonero, effective population size, genetic diversity, pedigree analysis, pedigree purging

## Abstract

**Background and Aim:**

Inbreeding is a major concern in livestock breeding because it can reduce genetic diversity, impair fitness, and compromise long-term population sustainability. Conventional pedigree-based inbreeding coefficients often fail to distinguish between recently accumulated inbreeding and historical ancestral inbreeding that may have undergone genetic purging. Consequently, evaluating both new and ancestral inbreeding provides a more comprehensive assessment of genetic risk. This study aimed to investigate the temporal dynamics of classical, new, and ancestral inbreeding coefficients in two native Venezuelan dairy cattle breeds, Carora and Criollo Limonero, and to compare their effective population sizes using multiple pedigree-based approaches.

**Materials and Methods:**

Pedigree records comprising 80,467 Carora cattle and 3,559 Criollo Limonero cattle born between 1984 and 2023 were analyzed. Classical, recursive, Ballou, Kalinowski (new and ancestral), and ancestral history coefficient (AHC) inbreeding estimates were calculated using GRain version 2.2 through gene-dropping simulations. Pedigree completeness, effective population size, regression analyses, and Pearson correlation analyses were performed to evaluate temporal trends, relationships among inbreeding estimators, and differences between breeds.

**Results:**

All pedigree-based inbreeding coefficients increased over time in both breeds, with ancestral inbreeding exhibiting the greatest increase. Using the AHC method, ancestral inbreeding increased from 0.26% to 8.59% in Carora cattle and from 0.13% to 4.17% in Criollo Limonero cattle. Regression analyses demonstrated significant temporal increases for all inbreeding measures (p < 0.001). Pearson correlation coefficients among inbreeding estimators ranged from 0.28 to 0.98 in Carora cattle and from 0.25 to 0.99 in Criollo Limonero cattle (p < 0.001), with the strongest associations observed between classical and new Kalinowski coefficients and between Ballou and AHC estimates. Effective population size varied substantially according to the estimation method, with ancestral Kalinowski estimates producing the largest values and AHC the smallest. Despite its considerably larger pedigree, the Carora breed exhibited consistently higher ancestral inbreeding than the Criollo Limonero breed.

**Conclusion:**

Pedigree purging approaches provided substantially greater insight into long-term genetic diversity than conventional pedigree inbreeding coefficients alone. The marked accumulation of ancestral inbreeding, particularly in the Carora breed, indicates that classical methods may underestimate genetic risk in native Venezuelan dairy cattle. Integrating ancestral and new inbreeding estimators into breeding and conservation programs could improve mating strategies, preserve genetic diversity, and support the long-term sustainability of locally adapted cattle populations. Future studies incorporating genomic information are warranted to validate pedigree-based evidence of genetic purging and optimize conservation management.

## INTRODUCTION

Within animal populations, inbreeding has traditionally been assessed using pedigree-based genealogical information to quantify the probability that two alleles at a locus are identical by descent (IBD) [1]. Advances in computational methods and pedigree analysis have substantially improved the estimation of inbreeding coefficients over recent decades. Among these developments are recursive algorithms that account for non-zero inbreeding in founders with unknown ancestry [2, 3] and computational approaches specifically designed to efficiently analyze large and complex pedigree datasets [4, 5]. These methodological improvements have enhanced the accuracy of pedigree analyses and facilitated the evaluation of genetic diversity in livestock populations with extensive genealogical records.

A major advancement in pedigree analysis has been the development of pedigree purging methodologies, which distinguish between ancestral and newly accumulated inbreeding by considering the historical and recent transmission of IBD alleles through common ancestors. These approaches include the methods proposed by Ballou [6], *Kalinowski et al.* [7], and Baumung *et al.* [8], which have been implemented in software packages such as GRain version 2.2 [8, 9]. Unlike conventional pedigree-based inbreeding coefficients, pedigree purging methods provide additional biological insight into the extent to which deleterious alleles may have been exposed to natural or artificial selection over successive generations. Ballou's approach estimates the cumulative proportion of the genome previously exposed to inbreeding, thereby reflecting long-term purging effects. In contrast, the method developed by Kalinowski *et al.* [7] partitions total inbreeding into ancestral and new components, allowing discrimination between historical genetic load and recently accumulated homozygosity. The ancestral history coefficient (AHC) further complements these estimators by quantifying the cumulative exposure of alleles to inbreeding throughout pedigree segregation, particularly in populations with incomplete or uneven pedigree depth. Collectively, these complementary approaches provide a more biologically meaningful evaluation of inbreeding dynamics than any single estimator alone, thereby improving the interpretation of long-term genetic processes in livestock populations.

Pedigree purging analyses have been successfully applied to several domestic animal species, including dog breeds that have experienced historical genetic bottlenecks [10]. However, studies involving native or indigenous cattle breeds remain limited. Published investigations have primarily focused on the indigenous Negra Andaluza cattle in Spain [11] and the German Angler and Red-and-White Dual-Purpose cattle breeds [12], where relatively weak correlations were observed between conventional pedigree inbreeding coefficients and pedigree purging estimators. Although ancestral inbreeding analyses have recently been reported for several European dairy breeds, comparable investigations in Latin American indigenous cattle remain scarce. In particular, few studies have applied the combined Ballou, Kalinowski, and AHC methodologies to Criollo cattle populations, and no previous study has directly compared a synthetic tropical dairy breed with a pure indigenous breed maintained under similar tropical production conditions using a unified pedigree purging framework.

**In Venezuela, two important native dairy cattle breeds have been developed:** the Criollo Limonero breed [13] and the Carora breed [14]. Despite their shared geographical origin, these breeds have followed markedly different demographic and breeding histories. The Criollo Limonero breed has persisted as a relatively small and geographically restricted population [15–17], whereas the Carora breed has achieved wider distribution and a substantially larger population, although its numbers remain considerably lower than those of widely used exotic dairy breeds [18–20]. Recent studies have demonstrated that the Criollo Limonero breed has experienced a substantial loss of genetic diversity, emphasizing the urgent need for effective conservation and reproductive management strategies because the breed is considered critically endangered [15]. Similarly, studies on the Carora breed have reported increasing levels of inbreeding accompanied by progressive erosion of genetic diversity, highlighting the necessity of implementing controlled mating programs to maintain long-term population viability [21]. Beyond their genetic significance, both breeds possess considerable economic, cultural, and environmental value because they are well adapted to tropical production systems and constitute an important component of Venezuela's livestock genetic heritage. Preserving their genetic diversity is therefore essential not only for maintaining sustainable dairy production but also for enhancing resilience to climate change, emerging diseases, and evolving production challenges.

Although previous studies have documented increasing inbreeding and declining genetic diversity in both Carora and Criollo Limonero cattle, they have relied predominantly on conventional pedigree-based inbreeding coefficients that do not distinguish between recently accumulated inbreeding and ancestral inbreeding that may have undergone genetic purging. Consequently, the relative contributions of historical and recent inbreeding to the current genetic structure of these populations remain poorly understood. Furthermore, no comprehensive study has simultaneously evaluated multiple pedigree purging estimators together with classical inbreeding coefficients and effective population size in native Venezuelan dairy cattle over an extended historical period. Such information is essential for accurately characterizing long-term genetic trends, identifying populations at greatest genetic risk, and developing evidence-based breeding and conservation strategies tailored to locally adapted cattle populations.

Therefore, this study aimed to comprehensively evaluate the temporal dynamics of classical, ancestral, and new pedigree-based inbreeding coefficients in the Carora and Criollo Limonero dairy cattle populations over a 40-year period (1984–2023). Specifically, the study compared conventional pedigree inbreeding coefficients with the pedigree purging approaches of Ballou, *Kalinowski*, and the AHC, examined the relationships among these complementary estimators, and estimated effective population size using multiple pedigree-based methodologies. We hypothesized that ancestral inbreeding would represent a substantially greater proportion of total inbreeding than recent inbreeding, particularly in the smaller and historically bottlenecked Criollo Limonero population. By integrating multiple pedigree-based approaches within a unified analytical framework, this study provides a more comprehensive assessment of long-term genetic diversity, pedigree purging, and population sustainability than has previously been available for native Venezuelan dairy cattle, thereby generating valuable information to support future breeding, conservation, and genetic management programs.

## MATERIALS AND METHODS

### Ethical approval

This study did not involve live animals, animal experimentation, animal handling, biological sample collection, or any invasive procedures. Therefore, approval from an Institutional Animal Care and Use Committee (IACUC) or other animal ethics committee was not required. The research was conducted exclusively using existing pedigree databases provided by the Asociación de Criadores de Ganado Carora (ASOCRICA), Venezuela, for the Carora cattle population and the National Institute for Agricultural Research (INIA), Venezuela, for the Criollo Limonero cattle population. Permission to access and use these pedigree records for scientific research was obtained from the respective database custodians. All pedigree information was anonymized before analysis, and no personally identifiable information relating to animal owners or breeders was accessed or reported. The study complied with the ethical principles for research using existing archival data and was conducted in accordance with institutional policies and internationally accepted standards for responsible research, data management, and scientific integrity.

### Study period and location

**Figure 1:**
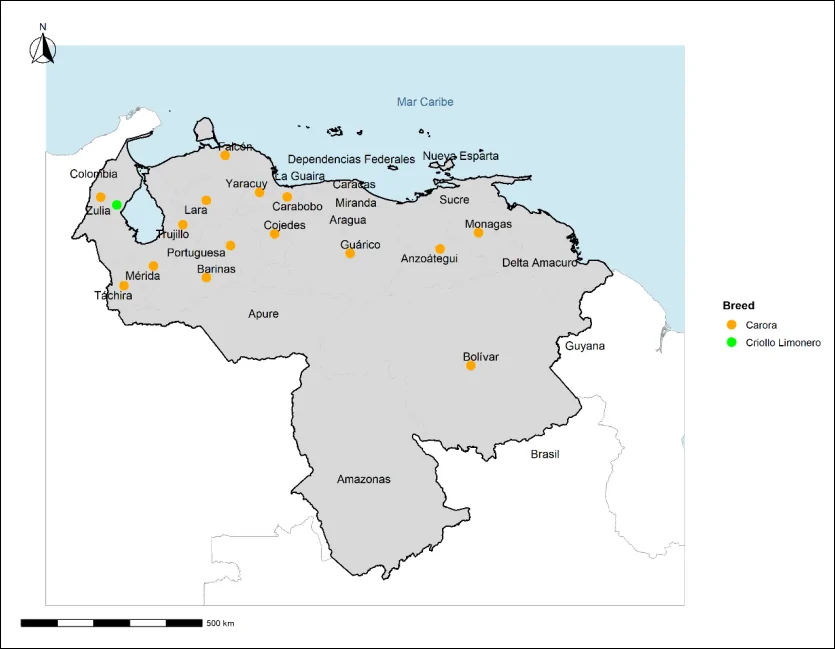
Geographical distribution of the native Venezuelan Carora and Criollo Limonero dairy cattle populations in Venezuela.

The data were extracted and analyzed from January to December 2024. The pedigree datasets included animals born over a 40-year period. Records for the Carora cattle population covered births from January 1984 to December 2023, whereas records for the Criollo Limonero cattle population extended from 1969 to 2023. The pedigree databases were obtained from ASOCRICA and INIA, both located in Venezuela. The geographical distribution of the native Venezuelan Carora and Criollo Limonero dairy cattle populations is presented in Figure 1.

### Study design

This study was designed as a retrospective pedigree-based population genetics analysis to evaluate classical, ancestral, and new inbreeding coefficients and effective population size in two native Venezuelan dairy cattle breeds. Pedigree completeness, multiple pedigree-derived inbreeding estimators, pedigree purging parameters, effective population size, regression analyses, and correlation analyses were evaluated to characterize long-term genetic diversity and inbreeding dynamics.

### Genealogical databases

**Figure 2:**
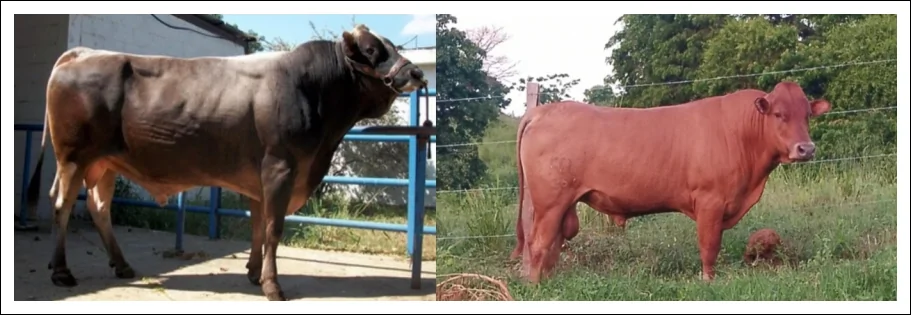
Native Venezuelan Carora and Criollo Limonero dairy cattle. Representative Carora sire (left) and Criollo Limonero sire (right).

The pedigree database of the Carora cattle population (Figure 2) was provided by the ASOCRICA, Venezuela. A total of 80,467 registered animals, comprising 8,067 bulls and 72,411 cows, born between January 1984 and December 2023, were included in the study. These records also incorporated genetic information from sires used through artificial insemination (AI). For the analyses, five populations were defined: one historical population consisting of all animals born between 1984 and 2023 and four decade-based subpopulations (1984–1993, 1994–2003, 2004–2013, and 2014–2023), comprising 8,444, 15,694, 34,987, and 21,350 individuals, respectively.

The pedigree database of the Criollo Limonero cattle population (Figure 2) was provided by the National Institute for Agricultural Research (INIA, Venezuela). The dataset consisted of 3,559 registered animals, including 1,178 bulls and 2,381 cows, born between 1969 and 2023. For comparative analyses, five populations were established, including the historical population (1969–2023) and four decade-based subpopulations (1984–1993, 1994–2003, 2004–2013, and 2014–2023), comprising 699, 460, 1,088, and 462 individuals, respectively. 

### Pedigree completeness index (PCI)

The PCI was calculated according to the methodology described by Navas *et al.* [22] using pedigree information from the first through the fifth ancestral generations. In addition, the number of maximum generations (GMax), complete generations (GCom), and equivalent complete generations (GEqu) were calculated as described previously [23].

Before analysis, pedigree records underwent comprehensive quality control to ensure pedigree integrity. Parentage conflicts, duplicate animal identification numbers, and incomplete ancestry records were carefully evaluated. Conflicting records were resolved by cross-checking birth dates, sex, and pedigree consistency, whereas duplicate records were merged or removed after verification. Animals with irreconcilable parentage information or missing critical ancestral records were excluded to minimize bias in the estimation of inbreeding coefficients and effective population size. Individuals with unknown parents were retained as founder animals to preserve pedigree structure without introducing artificial relationships.

### Pedigree inbreeding coefficients

Classical and recursive inbreeding coefficients were calculated according to the methodologies described by Wright [1], Sargolzaei *et al.* [5], and Aguilar *et al.* [3]. To investigate the contribution of historical ancestry to current inbreeding, pedigree purging methodologies were subsequently applied.

Ballou's ancestral inbreeding coefficient (fa_Ballou) is one of the most widely used pedigree-based measures for evaluating ancestral inbreeding and potential genetic purging [24, 25]. This coefficient represents the cumulative proportion of an individual's genome that has previously been exposed to inbreeding through its ancestors [6], thereby quantifying the historical accumulation of homozygosity over successive generations.

Kalinowski *et al.* [7] partitioned total inbreeding into two biologically distinct components: ancestral inbreeding (Fa_Kal), representing homozygous alleles that had already been IBD in previous generations, and new inbreeding (F_new), representing alleles becoming IBD for the first time. Because these components are estimated independently from the individual's total inbreeding coefficient, their values may differ substantially. Fa_Kal is equal to zero whenever the traditional inbreeding coefficient is zero because ancestral inbreeding depends on the presence of common ancestors shared through both paternal and maternal pedigree lines.

The AHC was also estimated. The AHC represents the expected number of times that a randomly selected allele has been IBD during pedigree segregation [8]. Kalinowski's inbreeding coefficients and the AHC were estimated using a gene-dropping simulation with 100,000 replicates.

The GRain package version 2.2 [8, 9] was used to calculate Ballou's ancestral inbreeding coefficient [6], the Kalinowski inbreeding coefficients [7], and the AHC defined by Baumung *et al.* [8]. Initially, the complete pedigree dataset was analyzed; subsequently, a pruned pedigree was generated to satisfy the assumptions required for the gene-dropping procedure implemented in GRain, which requires accurate and conflict-free pedigree information. This dual analytical strategy increased the robustness of comparisons between the substantially different Carora and Criollo Limonero populations by minimizing potential bias associated with pedigree depth and pedigree quality.

### Effective population size (Ne)

The effective population size (Ne) is defined as the number of breeding individuals in an idealized population that would produce the same rate of inbreeding observed in the actual population [26]. Initially, effective population size (Ne) was estimated from the regression coefficient (b) obtained by regressing the classical inbreeding coefficient (F) on the equivalent complete generation [27]. Subsequently, Ne was calculated using the following equation:


\[
Ne=\frac{1}{2b}
\]

The same approach was subsequently applied to estimate the ancestral effective population size, which reflects the rate of ancestral rather than total inbreeding. Accordingly, three additional estimates were obtained: Ne_Bal, Ne_Kal, and Ne_AHC, following the methodology described by Addo *et al.* [12].

### Statistical analysis

Pedigree analyses were performed using ENDOG version 4.8 [27], POPREP [28], RelaX2 [29], and the GRain package version 2.2 [8, 9], which were used to estimate demographic parameters, genetic diversity indices, gene origin probabilities, and pedigree-based inbreeding coefficients.

Relationships among pedigree inbreeding coefficients were evaluated using simple linear regression analyses with the classical, recursive, and pedigree purging estimators (f_classical, f_recursive, f_Bal, AHC, fa_Kal, and fnew_Kal) as dependent variables and year of birth as a continuous predictor. Prior to regression analyses, the assumptions of linearity, homoscedasticity, and normality of residuals were verified.

For each regression model, the regression coefficient, coefficient of determination (R²), coefficient of variation (CV), standard error of the mean (SEM), and corresponding p-value were calculated. Statistical significance was considered at p < 0.001. Because the analyses were exploratory, no adjustment for multiple comparisons was applied.

Effective population size was estimated using six pedigree-based approaches, including the classical (Ne_classical), recursive (Ne_recursive), Ballou (Ne_Bal), Kalinowski new (Ne_Kal-new), Kalinowski ancestral (Ne_Kal-a), and ancestral history coefficient (Ne_AHC) methods. Finally, Pearson correlation analyses were performed to evaluate relationships among the different pedigree-derived inbreeding coefficients. Unless otherwise specified, statistical significance was established at p < 0.05.

## RESULTS

## PCI

The pedigree completeness parameters of the Carora and Criollo Limonero cattle populations are presented in Table 1. Two contrasting patterns in pedigree completeness were observed between the two breeds. The historical Carora population comprised 80,467 registered animals, whereas the historical Criollo Limonero population included 3,559 registered animals, demonstrating the substantially larger pedigree size of the Carora breed.

**Table 1:** Pedigree completeness-derived parameters of the native Venezuelan Carora and Criollo Limonero dairy cattle populations.

**Parameter**	**Historical**	**1984-1993**	**1994-2003**	**2004-2013**	**2014-2023**
	**Carora / Limonero**	**Carora / Limonero**	**Carora / Limonero**	**Carora / Limonero**	**Carora / Limonero**
Pedigree records	80,467 / 3,559	7,716 / 699	14,610 / 460	32,994 / 1,088	25,147 / 462
No. generations (n)	18 / 16	10 / 9	12 / 11	15 / 14	18 / 16
1st generation (%)	60.21 / 88.69	63.95 / 87.27	56.68 / 90.43	51.08 / 94.90	69.11 / 90.69
2nd generation (%)	56.14 / 71.78	57.13 / 68.17	53.63 / 79.57	47.95 / 90.99	65.83 / 81.11
3rd generation (%)	52.00 / 54.63	47.72 / 35.05	50.74 / 60.90	45.92 / 81.54	63.61 / 78.17
4th generation (%)	47.77 / 40.38	38.48 / 18.33	46.39 / 40.33	44.19 / 66.65	62.01 / 68.93
5th generation (%)	43.66 / 26.59	31.38 / 7.74	40.66 / 22.91	42.10 / 45.49	60.51 / 57.39
Average GMax	9.45 / 7.10	4.72 / 4.38	7.35 / 7.42	9.49 / 10.12	12.07 / 11.70
Average GCom	1.33 / 1.70	0.97 / 1.34	1.17 / 1.72	1.18 / 2.34	1.73 / 2.15
Average GEqu	3.90 / 3.10	2.14 / 2.19	3.03 / 3.10	3.78 / 4.28	5.11 / 5.80

GMax: number of maximum generations; GCom: number of complete generations; GEqu: number of equivalent generations.

Regarding PCI, contrasting trends were observed between the historical populations of the two breeds from the first to the fifth ancestral generation. In the Carora population, pedigree completeness declined from 60.21% in the first generation to 43.66% in the fifth generation, whereas the corresponding values for the Criollo Limonero population decreased from 88.69% to 26.59%. The Criollo Limonero population therefore exhibited higher pedigree completeness in the first generation but a more pronounced reduction in pedigree completeness across subsequent generations than the Carora population.

Differences were also observed in pedigree depth parameters. The average GMax, GCom, and GEqu increased progressively across the evaluated periods in both breeds. During the 2014–2023 period, the Carora population showed average GMax, GCom, and GEqu values of 12.07, 1.73, and 5.11, respectively, whereas the corresponding values for the Criollo Limonero population were 11.70, 2.15, and 5.80. These results indicate progressive improvement in pedigree depth over time in both breeds.

### Pedigree purging-based measures

The pedigree-derived inbreeding coefficients estimated using the different methodologies are summarized in Table 2. Overall, all inbreeding coefficients showed an increasing trend across the study period in both breeds. The Carora population consistently exhibited higher inbreeding values than the Criollo Limonero population despite its considerably larger pedigree size.

Among the evaluated estimators, AHC produced the highest inbreeding values in both breeds. In the historical populations, AHC values were 5.16% in the Carora population and 1.72% in the Criollo Limonero population, increasing to 8.59% and 4.17%, respectively, during 2014–2023. Similarly, fa_Ballou increased progressively throughout the study period, reaching 7.84% in the Carora population and 3.98% in the Criollo Limonero population during the final evaluation period. In contrast, fa_Kalinowski consistently produced the lowest inbreeding estimates in both breeds, whereas fnew_Kalinowski showed intermediate values between the classical and ancestral estimators.

**Figure 3:**
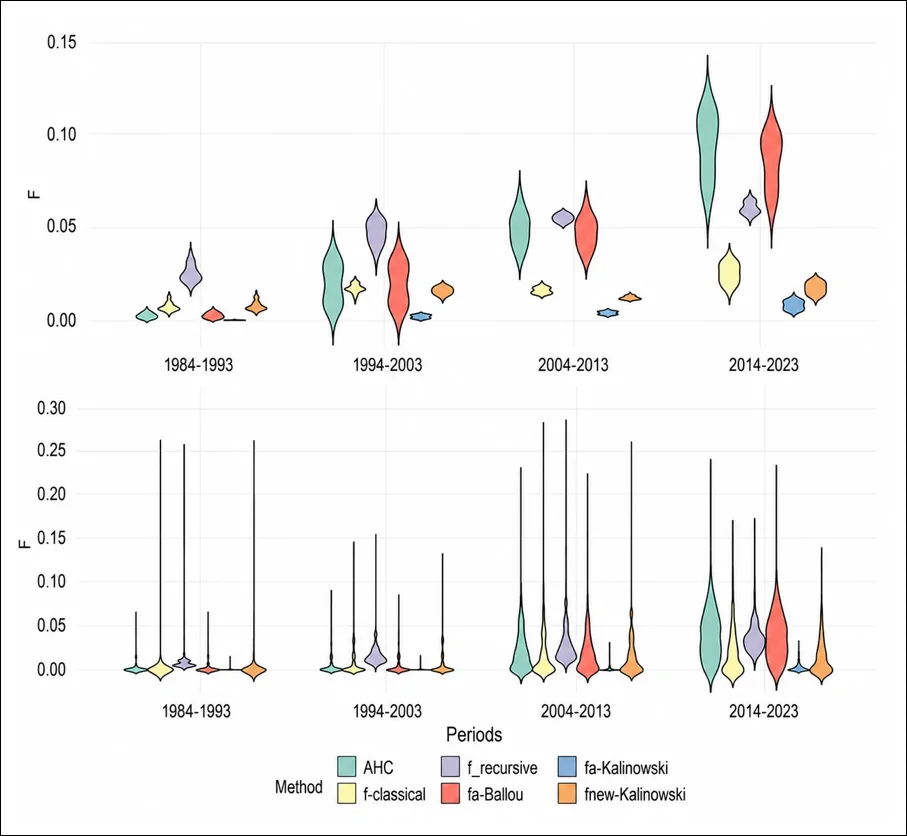
Violin plots showing the distribution of pedigree-derived inbreeding coefficients in the native Venezuelan Carora and Criollo Limonero dairy cattle populations. The upper panel represents the Carora population, and the lower panel represents the Criollo Limonero population. The distributions of f_recursive, f_classical, fa_Ballou, AHC, fnew_Kalinowski, and fa_Kalinowski were evaluated over the 40-year study period (1984–2023). All regression analyses were statistically significant (p < 0.001). AHC = Ancestral history coefficient

The evolution of pedigree-derived inbreeding coefficients is illustrated in Figure 3 using violin plots. These plots summarize the distribution of the inbreeding coefficients for each method by displaying the density distribution together with the median and interquartile range. Overall, the Carora population exhibited higher central values and wider distributions than the Criollo Limonero population, indicating greater variability in pedigree-derived inbreeding estimates.

**Table 2:** Pedigree-derived inbreeding coefficients estimated using different pedigree purging methods in the native Venezuelan Carora and Criollo Limonero dairy cattle populations.

**F Method**	**Historical**	**1984-1993**	**1994-2003**	**2004-2013**	**2014-2023**
	**Carora / Limonero**	**Carora / Limonero**	**Carora / Limonero**	**Carora / Limonero**	**Carora / Limonero**
n	80,467 / 3,559	7,716 / 699	14,610 / 460	32,994 / 1,088	25,147 / 462
f-classical	1.74 ± 0.01 /1.30 ± 0.05	0.78 ± 0.03 / 0.33 ± 0.06	1.68 ± 0.03 / 0.92 ± 0.09	1.61 ± 0.01 / 1.77 ± 0.08	2.25 ± 0.02 / 2.05 ± 0.12
f-recursive	5.16 ± 0.01 /2.55 ± 0.05	2.58 ± 0.03 / 1.03 ± 0.06	4.71 ± 0.02 / 2.08 ± 0.08	5.45 ± 0.01 / 3.08 ± 0.07	5.84 ± 0.01 / 4.08 ± 0.10
fa-Ballou	4.81 ± 0.01 /1.67 ± 0.05	0.26 ± 0.01 / 0.13 ± 0.02	2.07 ± 0.02 / 0.59 ± 0.06	4.77 ± 0.02 / 2.14 ± 0.07	7.84 ± 0.03 / 3.98 ± 0.13
AHC	5.16 ± 0.02 /1.72 ± 0.05	0.26 ± 0.01 / 0.13 ± 0.02	2.11 ± 0.02 / 0.59 ± 0.06	5.03 ± 0.02 / 2.18 ± 0.07	8.59 ± 0.03 / 4.17 ± 0.14
fa-Kalinowski	0.41 ± 0.00 /0.12 ± 0.01	0.01 ± 0.00 / 0.01 ± 0.00	0.18 ± 0.00 / 0.03 ± 0.01	0.38 ± 0.00 / 0.15 ± 0.01	0.72 ± 0.01 / 0.34 ± 0.02
fnew_Kalinowski	1.33 ± 0.01 /1.18 ± 0.04	0.76 ± 0.03 / 0.33 ± 0.06	1.50 ± 0.02 / 0.89 ± 0.09	1.23 ± 0.01 / 1.62 ± 0.07	1.54 ± 0.01 / 1.71 ± 0.10

AHC = Ancestral history coefficient

Distinct distributional patterns were observed among the different pedigree purging methods. The estimators separating ancestral and new inbreeding components showed different distribution patterns, reflecting differences between historical and recently accumulated inbreeding. Greater variability, characterized by wider distributions and more extreme values, was observed for all evaluated methods in the Criollo Limonero population than in the Carora population.

The AHC and fa_Ballou estimators showed the greatest concentration of inbreeding values from approximately 1995 onward in both breeds. In contrast, the classical and Kalinowski estimators exhibited more gradual increases during the same period. In the Carora population, AHC, f_recursive, and fa_Ballou consistently produced higher inbreeding estimates than the classical and Kalinowski methods. Conversely, in the Criollo Limonero population, the distributions of the different estimators showed minimal overlap.

### Temporal trends in pedigree-derived inbreeding coefficients

**Figure 4:**
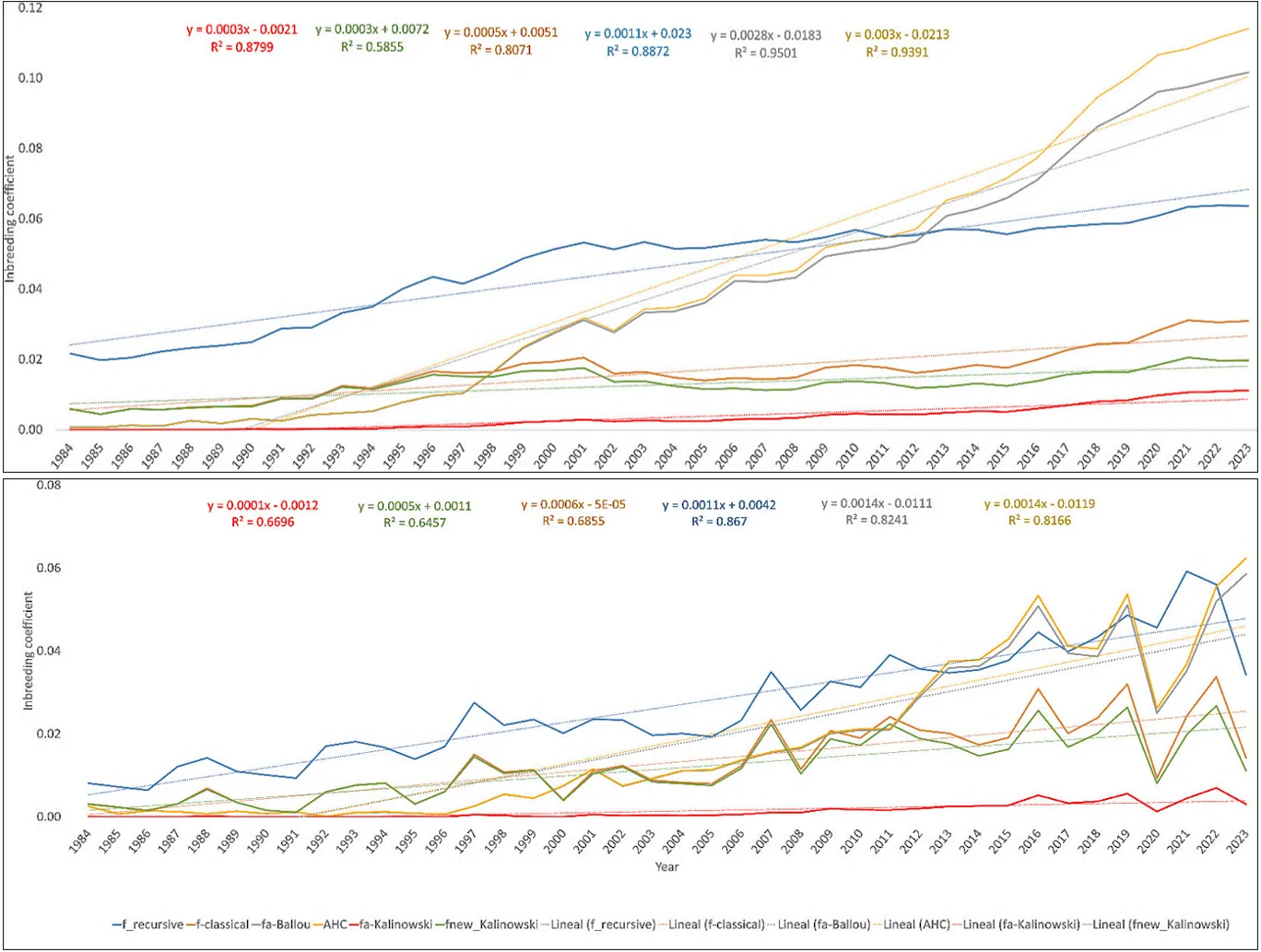
Temporal evolution of pedigree-derived inbreeding coefficients in the native Venezuelan Carora and Criollo Limonero dairy cattle populations over a 40-year period (1984–2023). The upper panel represents the Carora population, and the lower panel represents the Criollo Limonero population. Inbreeding coefficients were estimated using the f_recursive, f_classical, fa_Ballou, AHC, fnew_Kalinowski, and fa_Kalinowski methods. All regression analyses were statistically significant (p < 0.001). AHC = Ancestral history coefficient

The annual evolution of the pedigree-derived inbreeding coefficients is presented in Figure 4, and the corresponding regression equations and statistical parameters are summarized in Table 3.

**Table 3:** Regression equations and statistical parameters for pedigree-derived inbreeding coefficients in the Carora and Criollo Limonero dairy cattle populations.

**F Method**	**Equation**	**R² (%)**	**CV (%)**	**SEM**	**p-value**
Carora					
f_classical	y = 0.0051 + 0.0005x	80.71	19.37	0.0031	<0.001
f_recursive	y = 0.023 + 0.0011x	88.72	10.34	0.0048	<0.001
fa_Ballou	y = -0.0183 + 0.0028x	95.01	19.58	0.0075	<0.001
AHC	y = -0.0213 + 0.0030x	93.91	22.33	0.0092	<0.001
fnew_Kalinowski	y = 0.0072 + 0.0003x	58.55	21.34	0.0027	<0.001
fa_Kalinowski	y = -0.0021 + 0.0003x	87.99	34.21	0.0011	<0.001
Limonero					
f_classical	y = -0.0042 + 0.0006x	68.55	39.29	0.0051	<0.001
f_recursive	y = 0.0042 + 0.0011x	86.70	19.04	0.0050	<0.001
fa_Ballou	y = –0.0111 + 0.0014x	82.42	43.97	0.0075	<0.001
AHC	y = –0.0119 + 0.0014x	81.66	45.64	0.0081	<0.001
fnew_Kalinowski	y = 0.0011 + 0.0005x	64.57	38.63	0.0045	<0.001
fa_Kalinowski	y = –0.0012 + 0.0001x	66.96	75.24	0.0010	<0.001

CV = Coefficient of variation; SEM = Standard error of the mean; AHC = Ancestral history coefficient.

Most regression models showed strong temporal relationships, with R² values exceeding 80%. The only exception in the Carora population was f_classical (R² = 80.71%), whereas in the Criollo Limonero population, both fa_Kalinowski and fnew_Kalinowski exhibited R² values below 80%.

From 2011 onward, AHC and fa_Ballou exceeded f_recursive in the Carora population. In contrast, the Criollo Limonero population exhibited greater annual variability in all pedigree-derived inbreeding coefficients after 2014, with marked declines observed in 2020 and 2023.

Different temporal patterns were also observed among the estimators. In the Criollo Limonero population, f_recursive showed a greater increasing trend than AHC and fa_Ballou, whereas the opposite pattern was observed in the Carora population. Moreover, AHC, fa_Ballou, f_classical, and fnew_Kalinowski showed more pronounced declines during specific periods than f_recursive and fa_Kalinowski.

Three distinct groups of pedigree-derived estimators were identified in the Carora population: (1) AHC and fa_Ballou; (2) f_recursive; and (3) f_classical, fa_Kalinowski, and fnew_Kalinowski, which showed the lowest temporal trends. In the Criollo Limonero population, three different groups were identified: (1) f_recursive, AHC, and fa_Ballou; (2) f_classical and fnew_Kalinowski; and (3) fa_Kalinowski.

### Correlation among pedigree-derived inbreeding coefficients

Pearson correlation coefficients among the different pedigree-derived inbreeding estimators are presented in Table 4.

**Table 4:** Pearson correlation coefficients among pedigree-derived inbreeding estimators in the native Venezuelan Carora and Criollo Limonero dairy cattle populations.

**F Method 1**	**F Method 2**	**Correlation**	**CI (95%)**	**p-value**
AHC	fa_Ballou	0.99	0.998–0.998	<0.001
f_classical	fnew_Kalinowski	0.98	0.982–0.983	<0.001
f_classical	fa_Kalinowski	0.80	0.801–0.806	<0.001
f_recursive	fnew_Kalinowski	0.77	0.770–0.775	<0.001
f_classical	f_recursive	0.77	0.768–0.773	<0.001
AHC	fa_Kalinowski	0.71	0.705–0.712	<0.001
fa_Ballou	fa_Kalinowski	0.71	0.705–0.712	<0.001
fa_Kalinowski	fnew_Kalinowski	0.68	0.676–0.684	<0.001
f_recursive	fa_Kalinowski	0.57	0.566–0.576	<0.001
f_classical	fa_Ballou	0.44	0.432–0.444	<0.001
AHC	f_classical	0.43	0.427–0.438	<0.001
fa_Ballou	fnew_Kalinowski	0.32	0.314–0.327	<0.001
AHC	fnew_Kalinowski	0.31	0.307–0.320	<0.001
f_recursive	fa_Ballou	0.29	0.279–0.292	<0.001
AHC	f_recursive	0.28	0.278–0.291	<0.001

AHC = Ancestral history coefficient; CI = Confidence interval.

All pairwise correlations were statistically significant (p < 0.001). In the Carora population, the strongest correlation was observed between AHC and fa_Ballou (r = 0.99), followed by the correlation between f_classical and fnew_Kalinowski (r = 0.98). Moderate correlations were observed between f_classical and fa_Kalinowski and between f_recursive and fnew_Kalinowski, whereas weaker correlations involved the recursive estimator and the ancestral pedigree purging estimators.

In the Criollo Limonero population, the highest correlations were also observed between AHC and fa_Ballou (r = 0.99) and between f_classical and fnew_Kalinowski (r = 0.99). The lowest correlations involved f_recursive and the remaining pedigree-derived estimators, differing from the Carora population, in which f_recursive showed a relatively strong correlation with f_classical (r = 0.77). Overall, the correlations among the pedigree-derived inbreeding estimators were stronger in the Carora population than in the Criollo Limonero population.

### Effective population size (Ne)

The effective population size estimates derived from the different pedigree-based methods are presented in Table 5.

**Table 5:** Effective population size (Ne) estimated using different pedigree-derived inbreeding coefficients in the native Venezuelan Carora and Criollo Limonero dairy cattle populations.

**F Method**	**b**	**Ne**	**SEM**	**p-value**
Carora				
Ne_classical	0.0017	294.38	0.0001	<0.001
Ne_recursive	0.0009	563.69	0.0000	<0.001
Ne_Bal	0.0050	99.45	0.0001	<0.001
Ne_AHC	0.0059	84.64	0.0001	<0.001
Ne_Kal-new	0.0009	535.35	0.0001	<0.001
Ne_Kal-a	0.0008	654.01	0.0000	<0.001
Limonero				
Ne_classical	0.0007	735.63	0.0005	0.135
Ne_recursive	0.0018	454.94	0.0004	0.000
Ne_Bal	0.0010	521.57	0.0005	0.056
Ne_AHC	0.0011	283.19	0.0005	0.038
Ne_Kal-new	0.0005	1102.86	0.0004	0.234
Ne_Kal-a	0.0002	2209.21	0.0001	0.010

AHC = Ancestral history coefficient; CI = Confidence interval.

## DISCUSSION

### Pedigree completeness and pedigree structure

The present study evaluated the temporal evolution of classical, new, and ancestral inbreeding over approximately four decades in two native Venezuelan dairy cattle populations characterized by relatively small effective population sizes. A major strength of this study is its comprehensive analytical framework, which simultaneously evaluated classical and recursive inbreeding coefficients, three pedigree purging estimators, and six effective population size (Ne) estimators using the same pedigree datasets. To our knowledge, this is the first comprehensive pedigree-based comparison of classical, ancestral, and new inbreeding in the Carora and Criollo Limonero cattle populations and one of the most comprehensive pedigree purging analyses conducted in tropical dairy cattle.

The pedigree completeness of the Carora population was lower in the first and third ancestral generations (70.93%–65.62%) than that of the Criollo Limonero population (90.62%–78.17%). Nevertheless, the present findings indicate that population size alone did not determine pedigree completeness. Instead, pedigree recording practices and breeding management appear to have exerted a greater influence on pedigree integrity than the number of registered animals.

Limited pedigree completeness (low PCI) can substantially influence the partitioning of total inbreeding into ancestral and new components. Incomplete pedigrees truncate ancestral lineages, resulting in underestimation of ancestral inbreeding while simultaneously inflating estimates of new inbreeding. Such misclassification is particularly important in populations with incomplete or uneven pedigree records because it may exaggerate recent inbreeding while masking historical purging events. Consequently, pedigree completeness should always be considered when interpreting pedigree-derived estimates of ancestral and new inbreeding.

Comparable observations have been reported in other native cattle breeds. For example, the Berrenda en Negro (1,626 cows and 246 bulls) and Berrenda Colorada (2,651 cows and 523 bulls) breeds exhibited pedigree completeness exceeding 80% for the first and second ancestral generations [30]. These differences may reflect herd book regulations established by breed associations as well as the degree of breed openness resulting from open or closed herd book policies. Similarly, Guzerat cattle in Brazil [31] and Lithuanian dairy cattle [32] have reported pedigree completeness values exceeding 90%, largely attributed to the maintenance of closed herd books.

The Carora breed is currently managed using an open herd book designed to achieve breed purity through a grading-up (absorption) breeding program. This herd book comprises four registration categories: T5 (first-level crossbred), T4 (second-level crossbred), T3 (third-level crossbred), and T1 (pure Carora breed) (ASOCRICA, 2025). In contrast, the Criollo Limonero breed has been managed using a closed herd book within the principal conservation nuclei [15]. These contrasting breeding strategies likely contributed to the observed differences in pedigree completeness and pedigree depth between the two populations.

GEqu provide an estimate of the number of complete ancestral generations available for each individual. In the present study, GEqu reached 5.28 in the Carora population and 4.58 in the Criollo Limonero population. These values remain relatively low compared with those reported for other South American cattle breeds, including the Colombian Blanco Orejinegro breed (9.21) [33], although they are comparable to those reported for the Costeño con Cuernos (3.7), San Martinero (3.8), and Romosinuano (4.8) breeds [34].

Although pedigree recording for both Venezuelan breeds began during the 1960s, greater pedigree depth would be expected, particularly considering that the Colombian Blanco Orejinegro breed, whose pedigree recording began in 1980, has achieved substantially greater equivalent generations [33]. One possible explanation relates to differences in reproductive management. In the Carora population, breeding has relied on both natural mating and AI through a centralized semen distribution program. Although this system facilitates the widespread use of genetically superior sires, many breeding females may remain unregistered, or offspring may be entered into the herd book using only paternal information [18]. Such recording practices inevitably reduce pedigree completeness despite relatively large breeding populations.

In general, estimates of inbreeding and related pedigree parameters are influenced not only by breeding practices, including selection intensity, imported breeding animals, and AI, but also by pedigree completeness, the choice of founder population, and the methodology used to estimate inbreeding [35]. Incomplete pedigree information tends to underestimate true inbreeding; therefore, pedigree completeness values exceeding 0.60 have been recommended to obtain reliable pedigree-based inbreeding estimates [36, 37].

### Classical inbreeding in native Venezuelan cattle

The classical inbreeding coefficients estimated for the Carora and Criollo Limonero populations were 2.35% and 2.05%, respectively. These values were lower than those reported for the Negra Andaluza breed (7.23%) [38], the Berrenda Negra and Berrenda Colorada breeds in Spain (7.0% and 5.7%, respectively) [30], and the Reyna breed of Nicaragua (4.5%–11.2%) [39, 40].

Within Latin America, the estimated inbreeding coefficients were comparable with or slightly higher than those reported for several native cattle breeds, including the Criollo Limonero breed of Venezuela (2.05%) [15], Costeño con Cuernos (0.79%), San Martinero (0.87%), Blanco Orejinegro (0.18%–2.88%), and Romosinuano (1.22%–2.53%) cattle in Colombia [33, 41]. However, they remained lower than those reported for the Mexican Romosinuano (3.7%) and Lechero Tropical (3.48%) breeds [34, 42, 43].

The marked differences between Iberian and Latin American native breeds may largely reflect differences in pedigree depth. European breeds generally possess deeper and more complete genealogical records, whereas pedigree incompleteness in many Latin American breeds may lead to underestimation of classical inbreeding coefficients. Differences in breeding structure, including the number of breeding sires used and reproductive management practices, may also contribute to these contrasting estimates, as previously demonstrated for the Negra Andaluza breed in Spain [38] and the Reyna breed in Nicaragua [40].

Despite the substantial difference in census population size between the Carora and Criollo Limonero populations, their classical inbreeding coefficients were remarkably similar. In the Criollo Limonero population, the relatively small breeding population and limited number of breeding animals likely contribute to the accumulation of inbreeding. Conversely, the larger Carora population benefits from widespread use of AI, allowing a greater number of females to participate in breeding programs and thereby slowing the rate of inbreeding accumulation despite intensive selection [44].

Interestingly, ancestral inbreeding was consistently higher in the Carora population than in the Criollo Limonero population. This finding may reflect differences in the efficiency of genetic purging between the two breeding systems. Intensive directional selection combined with extensive use of AI in the synthetic Carora breed may have increased the exposure of deleterious recessive alleles to selection, thereby facilitating their gradual removal from the population. In contrast, the closed herd book maintained in the Criollo Limonero population may have resulted in different purging dynamics because of its smaller breeding population and more restricted gene flow. These contrasting patterns may have important implications for maintaining adaptation to tropical environments, improving resilience to heat stress, and enhancing the long-term sustainability of locally adapted cattle populations under future climate change scenarios.

### Ancestral inbreeding and pedigree purging

Studies evaluating ancestral inbreeding in both indigenous and commercial cattle breeds remain limited. In the present study, ancestral inbreeding estimated using pedigree purging methods increased progressively over time in both the Carora and Criollo Limonero populations, with the Ballou and AHC estimators producing the highest values. Similar trends have been reported for the German Brown Swiss breed by Wirth *et al.* [45], supporting the usefulness of pedigree purging approaches for evaluating the historical accumulation of inbreeding.

Ballou [6] defined ancestral inbreeding as the cumulative proportion of an individual's genome that has previously been exposed to inbreeding in its ancestors. Consistent with this concept, the Ballou estimator showed marked increases during the last two evaluation periods in both breeds. In the Carora population, fa_Ballou increased from 5.05% to 8.22%, whereas in the Criollo Limonero population it increased from 2.14% to 3.98%. These values were higher than those reported for the Negra Andaluza breed (1.98%) [11], German Brown Swiss (2.2%) [45], German Angler (3.69%), Red-and-White Dual-Purpose cattle (1.39%) [12], and Irish Holstein cattle (6.50%–6.89%) [47], but were comparable with those reported for German Holstein cattle (8.15%) [46].

The progressive increase in ancestral inbreeding observed in both Venezuelan breeds indicates that a considerable proportion of homozygous genomic regions has persisted over multiple generations. Such accumulation reflects the long-term demographic history of these populations and highlights the importance of complementing conventional inbreeding coefficients with pedigree purging estimators when evaluating genetic diversity. Unlike classical inbreeding coefficients, ancestral inbreeding provides additional information regarding the historical exposure of deleterious alleles to selection and the potential occurrence of genetic purging.

### Biological significance of ancestral and new inbreeding

The biological consequences of ancestral and new inbreeding have been investigated in several livestock species, although reported effects remain variable [6, 48]. In cattle, recent (new) inbreeding is generally considered more detrimental to economically important traits, including production, fertility, and reproductive performance [47, 49–51]. In contrast, ancestral inbreeding has been associated with both unfavorable effects [52] and beneficial consequences resulting from the gradual purging of deleterious recessive alleles [46].

Evidence supporting genetic purging has also been reported in sheep, dogs, rabbits, and goats [53–58]. Nevertheless, despite the potential reduction in genetic load through purging, the continuous accumulation of inbreeding remains a major concern because of its adverse effects on long-term genetic diversity, population fitness, and economic performance [59, 60]. Therefore, both ancestral and new inbreeding should be considered jointly when designing breeding and conservation strategies, particularly for small or genetically vulnerable livestock populations.

### Effective population size and implications for conservation

The effective population size (Ne) estimates obtained in the present study were generally lower than those previously reported for the Criollo Limonero [15] and Carora [21] populations. Considerable variation among estimation methods was also observed, highlighting the sensitivity of Ne estimation to the pedigree-derived inbreeding coefficient used.

The relatively low Ne_recursive estimate observed for the Carora population likely reflects the sensitivity of recursive algorithms to incomplete pedigree information rather than the actual demographic structure of the population. Recursive algorithms propagate the effects of unknown parents and missing ancestral information, thereby increasing estimated coancestry and reducing Ne estimates. Consequently, in populations characterized by widespread use of AI and a relatively broad breeding base, such estimates may underestimate the true effective population size because of methodological limitations rather than genuine demographic decline.

Although the Ballou and AHC estimators also produced relatively low Ne estimates, values obtained using the Kalinowski estimator were comparable with those reported previously. Similar methodological differences have been described for the German Angler and Red-and-White Dual-Purpose cattle breeds [12]. These discrepancies probably arise because Ne is traditionally estimated using Wright's classical inbreeding coefficient (F), which assumes an ideal population affected only by finite population size and neutral evolutionary processes. Applying alternative pedigree purging coefficients directly to the classical Ne equation may violate these assumptions and generate biologically unrealistic estimates.

Furthermore, estimation of purged inbreeding is inherently more complex because it depends on the effective purge coefficient (d = 0.5s[1−2h]), which is generally unknown and is frequently estimated simultaneously with the purged inbreeding coefficient itself. Consequently, interpretation of Ne estimates derived from pedigree purging methods should be performed with caution.

An additional consideration is that genetic purging is a gradual evolutionary process requiring multiple generations before measurable effects become apparent. Reliable inference regarding purging therefore depends on sufficiently deep and complete genealogical records extending from the establishment of the population or, more precisely, from the historical bottleneck. The minimum number of generations required depends largely on effective population size. Under completely recessive inheritance, the lower limit can be approximated by tc = √(2Ne) [61]. For example, a population with Ne = 50 would require approximately 10 generations before the effects of purging become detectable. Consequently, pedigree-based measures of purging should not be interpreted as direct evidence of purging unless sufficient time has elapsed for these evolutionary processes to occur.

## CONCLUSION

This study provides the first comprehensive pedigree-based comparison of classical, ancestral, and new inbreeding coefficients in the native Venezuelan Carora and Criollo Limonero dairy cattle populations using multiple pedigree purging methodologies. Although both breeds exhibited relatively low classical inbreeding coefficients, all pedigree-derived estimators demonstrated a progressive increase in inbreeding over time, particularly the ancestral components estimated using the Ballou and AHC methods. Despite its considerably larger population size, the Carora breed showed greater ancestral inbreeding than the Criollo Limonero breed, indicating that conventional pedigree-based inbreeding coefficients alone may underestimate the historical accumulation of homozygosity and the underlying genetic risk.

The integration of multiple pedigree purging estimators with effective population size analyses represents a major strength of this study, providing a more comprehensive understanding of long-term genetic diversity than conventional pedigree analyses alone. These findings emphasize the importance of incorporating ancestral and new inbreeding measures into routine genetic monitoring programs to improve mating decisions, minimize the accumulation of harmful homozygosity, and support the sustainable conservation and management of locally adapted cattle breeds.

This study has some limitations. The analyses were based exclusively on pedigree records, and the accuracy of the estimated inbreeding coefficients depends on pedigree completeness and recording quality. Furthermore, pedigree-based analyses cannot directly identify genomic regions affected by selection or quantify the actual genetic load underlying inbreeding depression.

Future studies should integrate pedigree information with genomic approaches, including runs of homozygosity (F_ROH), ancestral runs of homozygosity, whole-genome analyses, and associations with economically important production, fertility, adaptation, and fitness traits. Such integrated analyses will provide direct evidence of genetic purging and improve the development of breeding and conservation strategies for native tropical cattle populations.

Overall, the findings demonstrate that pedigree purging methodologies provide substantially greater insight into the historical dynamics of inbreeding than classical pedigree-based approaches alone. Incorporating ancestral and new inbreeding estimators into conservation and breeding programs will facilitate more informed genetic management, promote the preservation of genetic diversity, and enhance the long-term sustainability and resilience of the Carora and Criollo Limonero dairy cattle populations.

## DATA AVAILABILITY

The data generated during the study are included in the manuscript.

## GENERATIVE AI DECLARATION

Quality of Figures 3 and 4 have been improved with the help of ChatGPT Plus. The authors declare that no generative artificial intelligence or AI-assisted technologies were used in the writing, analysis, or preparation of this manuscript. 

## AUTHORS’ CONTRIBUTIONS

LFCM: Conceptualization, study design, methodology, supervision, coordination of the study, investigation, data curation, formal and statistical analyses, validation, and writing the original draft. JRPG, EJJQ, and AGLG: Conceptualization, data collection, data curation, investigation, validation, and review and editing of the manuscript. JGF: Conceptualization, investigation, experimental assistance, validation, and review and editing of the manuscript. MGH: Conceptualization, study design, supervision, coordination of the study, investigation, formal analysis, validation, and writing the original draft. All authors have read and approved the final manuscript.
